# Fouling Resistant CA/PVA/TiO_2_ Imprinted Membranes for Selective Recognition and Separation Salicylic Acid from Waste Water

**DOI:** 10.3389/fchem.2017.00002

**Published:** 2017-01-26

**Authors:** Xiaopeng Yu, Xueyang Mi, Zhihui He, Minjia Meng, Hongji Li, Yongsheng Yan

**Affiliations:** ^1^Jilin Provincial Key Laboratory for Numerical Simulation, Jilin Normal UniversitySiping, China; ^2^School of Computer Science, Jilin Normal UniversitySiping, China; ^3^School of Chemistry and Chemical Engineering, Jiangsu UniversityZhenjiang, China

**Keywords:** salicylic acid, cellulose acetate, titanium oxide nanoparticles, phase inversion, imprinted membranes

## Abstract

Highly selective cellulose acetate (CA)/poly (vinyl alcohol) (PVA)/titanium dioxide (TiO_2_) imprinted membranes were synthesized by phase inversion and dip coating technique. The CA blend imprinted membrane was synthesized by phase inversion technique with CA as membrane matrix, polyethyleneimine (PEI) as the functional polymer, and the salicylic acid (SA) as the template molecule. The CA/PVA/TiO_2_ imprinted membranes were synthesized by dip coating of CA blend imprinted membrane in PVA and different concentration (0.05, 0.1, 0.2, 0.4 wt %) of TiO_2_ nanoparticles aqueous solution. The SEM analysis showed that the surface morphology of membrane was strongly influenced by the concentration of TiO_2_ nanoparticles. Compared with CA/PVA-TiO_2_(0.05, 0.1, 0.2%)-MIM, the CA/PVA-TiO_2_(0.4%)-MIM possessed higher membrane flux, kinetic equilibrium adsorption amount, binding capacity and better selectivity for SA. It was found that the pseudo-second-order kinetic model was studied to describe the kinetic of CA/PVA-TiO_2_(0.2%)-MIM judging by multiple regression analysis. Adsorption isotherm analysis indicated that the maximum adsorption capacity for SA were 24.43 mg g^−1^. Moreover, the selectivity coefficients of CA/PVA-TiO_2_ (0.2%)-MIM for SA relative to *p*-hydroxybenzoic acid (*p*-HB) and methyl salicylate (MS) were 3.87 and 3.55, respectively.

## Introduction

Salicylic acid (SA) is a well-known prominent active pharmaceutical ingredient (API) in various pharmaceutical preparations, especially in the preparation of anti-inflammatory drug acetylsalicylic acid (aspirin; Huang et al., 2013). It is also an important ingredient of cosmeceutical to unclog the pores, lighten the stain and reduce wrinkles (Jafari et al., 2012). However, on account of the poor technology of separation in the SA industrial production, it has been created a great deal of SA-contained industrial wastewater. Recently, SA has been identified as a water pollutant due to its chroma and high ecotoxicity, and it has been detected in sewage and even drinking water, which will damage to liver and kidney function, inducing protein denaturation and even causing the mucosal bleeding (Meng et al., 2013). Additionally, the high concentration of SA in SA-contained wastewater is difficult to be disposed by the general sewage treatment technology, which has resulted in losing a large number of valuable SA. Therefore, it is of great significance in developing efficient methods for selectively separating SA from SA-contained water.

In decade years, the molecular imprinting technology (MIT; Sellergren, 2000; Pan et al., 2011; Chen et al., 2015; Wang et al., 2016) has been used to introduce specific molecular recognition properties into molecularly imprinted membranes (MIMs; Zhang et al., 2011; Donato et al., 2013). MIM technology has made some advances in selectively separating target molecules from solutions by simple membrane permeation processes (Puoci et al., 2012; Wu et al., 2014a; He et al., 2015; Wang et al., 2015) such as low energy, simple device. However, the application of conventional MIM that obtained by surface photo-grafting polymerization is limited because of the drawbacks such as time-consuming, complex procedures, and high material loses in membrane preparation.

Recently, phase inversion technique is a new innovative way for the synthesis of MIM due to the simplicity of the processes. By using phase inversion method, the polymer matrices and functional polymer are dissolved in solvent containing template molecules. Then, interactions take place between the template molecules and the functional groups of polymer. The binding sites and membrane morphology are simultaneously formed when the casting solution coagulates in the non-solvent (Del Blanco et al., 2012; Dima et al., 2013). At present, various membrane materials, such as cellulose acetate (CA; Zafar et al., 2012), polyethersulfone (PES; Ahmad et al., 2013), polysulfone (PS; Peng et al., 2013), polyvinylidene fluoride (PVDF; Zhao et al., 2012), have been used in phase inversion technique. And various polymers, such as PES (Han et al., 2014), PBI (Székely et al., 2015), ANAA (Wang et al., 1996), AN (Cheku et al., 2008), have been used in the phase inversion for imprinted membranes. Compared with multiple membrane properties, CA membranes are suitably used in both water and wastewater treatments (Abedini et al., 2011; Ghaemi et al., 2012; Rana et al., 2012). However, the application of pure CA membrane is limited due to the insufficient hydrophilicity and low antifouling performance. As mentioned above, the drawbacks have been recognized as the main barriers for the further development of the CA membrane. Therefore, the aim of the present study is to develop the CA membrane with high antifouling performance and excellent molecular imprinting selectivity.

Recently, MIT has been used as promising method for waste water treatment (Puoci et al., 2012; He et al., 2015; Luo et al., 2015; Razali et al., 2015). In the context of these progress, porous CA blend imprinted membranes (CA-MIMs) were firstly synthesized here by phase inversion method with CA as the polymer matrice, polyethyleneimine (PEI) as the functional polymer, and the salicylic acid (SA) as the template molecule. Then, the CA-MIMs were modified with poly(vinyl-alcohol) (PVA) and nano-crystalline titanium oxide (TiO_2_, titania) nanoparticles to improve the hydrophilic and antifouling properties of MIMs. Finally, the performance of TiO_2_ nanoparticles concentration on the surface of CA composite membrane was discussed by membrane morphology, membrane flux, isothermal adsorption, selective adsorption, and transport-selectivity permeation.

## Experimental

### Materials

Cellulose acetate (CA), salicylic acid (SA), *p*-hydroxybenzoic acid (*p*-HB), methyl salicylate (MS), and dimethyl sulfoxide (DMSO) were obtained from Sinopharm Chemical Reagent (Shanghai, China). Isocyanates and Polyethyleneimine (PEI) (Mn = 70000), 50 wt % aqueous solution from Aladdin reagent was used as such. Nano-sized TiO_2_ (25 nm), poly(vinyl-alcohol) (PVA, 98% alcoholysis), and glutaraldehyde (GA) 25% (w/w) were also purchased from Sinopharm Chemical Reagent (Shanghai, China). Ultrapure water was used throughout this study.

### Synthesis of membranes

#### Synthesis of CA imprinted and non-imprinted membranes

**Figure 1:**
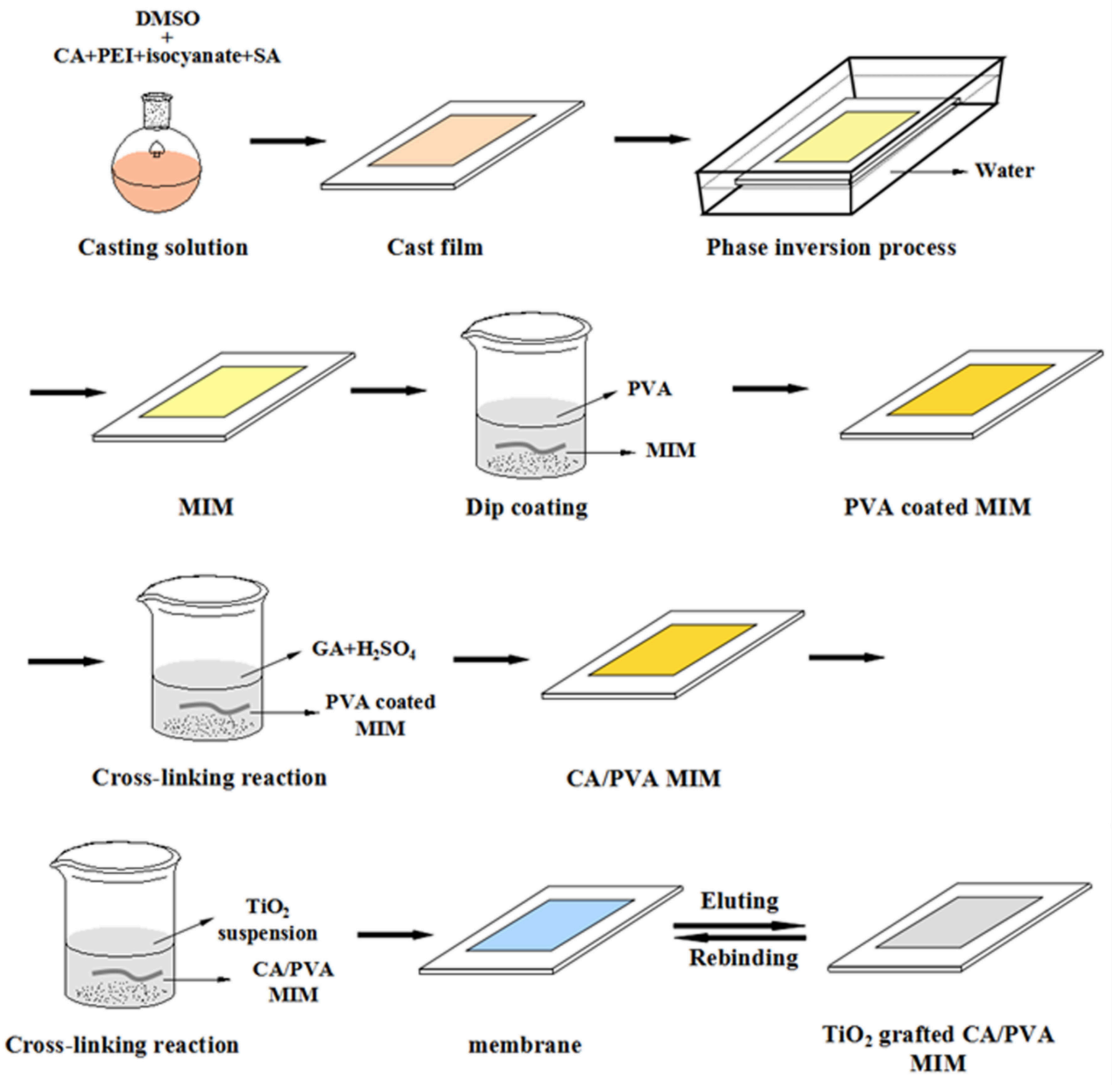
**Schematic illustration of preparing TiO_**2**_ grafted CA/PVA molecularly imprinted membrane**.

The blend imprinted membranes were fabricated via phase inversion method, as shown in Figure 1. The homogeneous solution was prepared by dissolving CA, 50 wt % PEI and isocyanates with template SA by stirring for 4.0 h at 40°C. The total polymer concentration of casting solution was 13 wt % in DMSO as the solvent. The mass ratio of the CA/50 wt % PEI/ isocyanates was 10/1.5/0.75 and the content of SA in casting solution was ~0.3 wt %. Then, the blended PEI was crosslinked by isocyanates in the same solvent under magnetic stirring. After formation of a homogeneous solution, the casting solution was hold for around 24 h to remove the air bubbles. Then, the casting solution (0.45 mm) was cast on a polished glass substrate under ambient conditions. After 30 s the cast film was quickly immersed into the non-solvent (water) at least 1.0 h for removing most of the solvent and water-soluble polymer. The formed membrane sheet was subsequently peeled-off and preserved in deionized water. The blend imprinted membranes (MIM) was extracted with mixed solvents of methanol and acetic acid (9:1, v/v) in a Soxhlet apparatus to remove the templates SA. Additionally, the blend non-imprinted membrane (NIM) was prepared in the same procedure but without the templates.

#### Synthesis of PVA coated CA membranes

The homogeneous PVA solution was obtained by dissolving 1.5 wt % of PVA in water by mechanical stirring (24 h at 90°C). After that, the PVA was coated on the surface of CA membrane via dip coating technique. To reduce the membrane swelling, the PVA coated CA membranes were immersed in the cross-linking solution containing 5.0 wt % of GA and 0.5 wt % of H_2_SO_4_ for 2.0 min at room temperature. H_2_SO_4_ was the catalyst of the chemical cross-linking reaction. The reaction process of PVA with GA was shown in Figure 1.

#### Synthesis of TiO_2_ grafted CA membranes

The whole preparation process of TiO_2_ grafted CA imprinted membrane was shown in Figure 1. Firstly, TiO_2_ nanoparticles suspensions were prepared by dispersing various concentrations (0.05, 0.1, 0.2, 0.4 wt %) of TiO_2_ nanoparticles in deionized water. To avoid the agglomeration of nanoparticles, the TiO_2_ suspensions were stirred vigorously for 60 min with sonication. Then, the PVA coated CA membranes were immersed in the TiO_2_ nanoparticles suspensions with continuous shaking for 1.0 day at 25°C. Afterwards, the TiO_2_ grafted CA composite membranes were immersed in deionized water using tip ultrasound equipment for 2.0 h to remove the non-grafted nanoparticles. Finally, all CA/PVA coated with TiO_2_ membranes (CA/PVA-TiO_2_(0.05%), CA/PVA-TiO_2_(0.1%), CA/PVA-TiO_2_(0.2%), and CA/PVA-TiO_2_(0.4%) were washed with water at least three times and preserved in deionized water before tests.

### Characterization

The surface micrographs of pure CA and its TiO_2_ grafted CA composite membranes were observed by scanning electron microscopy (SEM, S-4800). Before photographing, all the prepared membranes were cleaned with deionized water and dried in vacuum then fractured. The dried membrane samples were gold sputtered for producing electric conductivity, and were operated with the microscope.

### Membrane flux experiments

The membrane was fitted on the ultrafiltration cell (UF-8010, Amicon) with the effective membrane area 4.9 cm^2^. A aqueous solution containing 50 mg L^−1^ SA was prepared as the feed solution to measure the membrane flux. The feed solution permeated through different membranes and the flux of the different membranes could be calculated by Equation (1):

(1) $$\begin{array}{l} J=\frac{V}{st} \end{array}$$

where *J* is the flux of the membrane (mL cm^−2^ min^−1^), *V* is the volume of permeate solution (mL), *t* and *s* are the operation time (min) and effective area of the membrane (cm^2^), respectively.

### Batch mode binding experiments

The adsorption kinetics experiments were carried out to determine the adsorption equilibrium time and rate-limiting factor of prepared CA membranes for the template SA. The prepared membranes were placed in 10 mL centrifuge tubes containing 9.0 mL of SA methanol-water solutions (100 mg L^−1^). These mixtures were shaken on a constant temperature shaker. At different points of time, the concentration of SA in the solutions were obtained and determined by UV spectrophotometer at a wavelength of 303 nm. The binding amounts (*q*, mg g^−1^) were determined by the following Equation (2), and the time of adsorption equilibrium was obtained.

(2) $$\begin{array}{l} q_{\text{e}}=\frac{\left(C_{0}-C_{t}\right)V}{W} \end{array}$$

where *C*_0_ and *C*_t_ (mg L^−1^) are the feed concentration at the initial time and the sampling time, respectively. *V* and *W* are the volume of the solution (mL) and weight of the prepared membrane (g), respectively.

The batch binding experiments were performed in 10 mL centrifuge tubes containing prepared membrane and contained 9.0 mL SA methanol-water solution with different concentrations ranging from 25 to 300 mg L^−1^. These centrifuge tubes were placed in 25°C water bath for 3.0 h. After reaching adsorption equilibrium, the residual concentrations of SA in the solution were determined by UV spectrophotometry. Then the equilibrium binding amounts (*q*_e_, mg g^−1^) were calculated with Equation (3):

(3) $$\begin{array}{l} q_{\text{e}}=\frac{\left(C_{0}-C_{e}\right)V}{W} \end{array}$$

where *C*_0_ and *C*_t_ (mg L^−1^) are the feed concentration at the initial time and the saturated binding time, respectively. *V* (mL) and *W* (g) are the volume of the solution and the weight of the prepared membrane, respectively.

### Selective recognition experiments

To investigate the selective properties of SA, both *p*-HB and MS were selected as the similar compounds. A piece of membrane was added into a 10 mL centrifuge tube, each of which contained 9.0 mL the coexisting compound solution with 15 mg L^−1^ of SA and contrast substance (*p*-HB or MS), respectively. After adsorption, the concentration of substrate (SA, *p*-HB, MS) in the solution were determined by UV spectrophotometry and the binding amounts of membrane for SA and the analogs were calculated as the procedure of the batch binding experiments.

The distribution coefficient (*K*_d_) and selectivity coefficient (α) of *p*-HB and MS with respect to SA were obtained according to Equations (4, 5):

(4) $$\begin{array}{l} K_{\text{d}}=\frac{q_{e}}{C_{e}} \end{array}$$

*K*_d_ (mL g^−1^) is the distribution coefficient, *q*_e_ (mg g^−1^) and *C*_e_ (mg L^−1^) are the equilibrium binding amount and the equilibrium concentration of adsorption in solution, respectively.

The selectivity coefficient (α) was obtained according to the following equation:

(5) $$\begin{array}{l} \alpha =\frac{K_{\text{di}}}{K_{\text{dj}}} \end{array}$$

where *i* and *j* represent the template and the competitive analog, respectively.

### Antifouling experiments

The CA/PVA-TiO_2_(0.2%)-MIM was subjected to a 600 ppm BSA solution to evaluate its antifouling properties. The BSA solution flux was calculated nine times in 3 h. The rate of magnetic stirrer was set to 300 rpm to reduce the effect of concentration polarization of CA/PVA-TiO_2_(0.2%)-MIM. And then the BSA solution was replaced with distilled water, and the rate of magnetic stirrer was set to 500 rpm for 30 min. The flux recovery ratio (FRR) of the CA/PVA-TiO_2_(0.2%)-MIM was calculated by the following equation:

(6) $$\begin{array}{l} FRR=\left(\frac{J_{wa}}{J_{wb}}\right)\times 100 \end{array}$$

where *J*_*wb*_ and *J*_*wa*_ (mL cm^−2^ min^−1^) are the pure water fluxes of the CA/PVA-TiO_2_(0.2%)-MIM before and after BSA solution filtration. The formation of fouling layer on the surface of CA/PVA-TiO_2_(0.2%)-MIM and concentration polarization will cause the reversible fouling that can be reversed (cleaned) by washing using distilled water. The reversible fouling (RF_*r*_) was calculated by the following equation:

(7) $$\begin{array}{l} RF_{r}=\left(\frac{J_{wa}-J_{BSA}}{J_{wb}}\right)\times 100 \end{array}$$

Where *J*_*BSA*_ (mL cm^−2^ min^−1^) was the BSA soultion flux of the CA/PVA-TiO_2_(0.2%)-MIM. However, the adsorption on the surface of the CA/PVA-TiO_2_(0.2%)-MIM cannot be cleaned (reversed) by distilled water washing, and it only can be cleaned by chemical washing. This was named irreversible fouling that was calculated by the following equation:

(8) $$\begin{array}{l} RF_{ri}=\left(\frac{J_{wb}-J_{wa}}{J_{wb}}\right)\times 100 \end{array}$$

## Results and discussion

### Scanning electron microscopy (SEM) analysis

**Figure 2:**
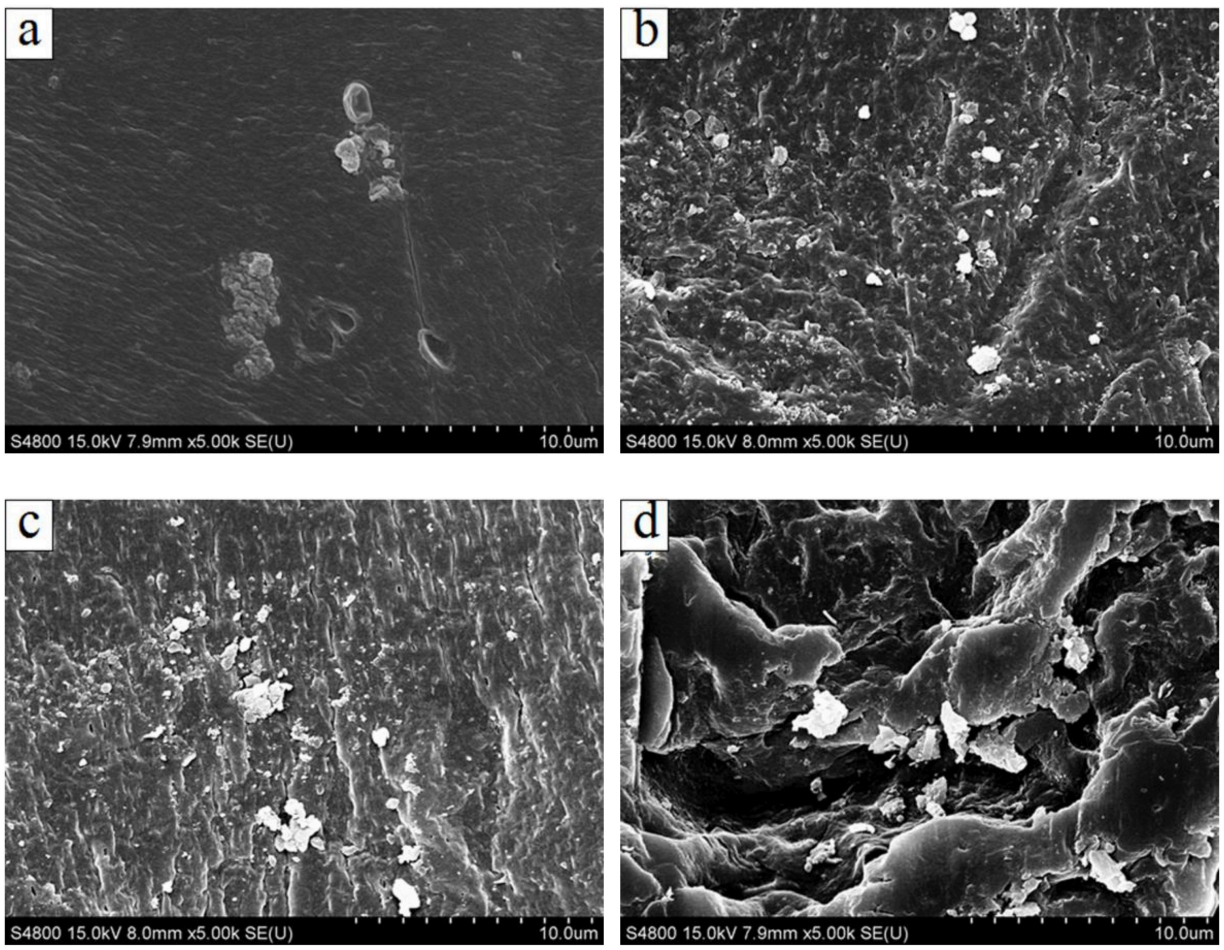
**The surface SEM image of PVA/CA composite modified imprinted membranes with TiO_**2**_ nanoparticles: (A)** 0.05 wt % TiO_2_; **(B)** 0.1 wt % TiO_2_; **(C)** 0.2 wt % TiO_2_; **(D)** 0.4 wt % TiO_2_.

Figure 2 shows the surface morphologies of PVA/CA composite modified imprinted membranes with TiO_2_ nanoparticles. It can be observed that there is an amount of TiO_2_ nanoparticles grafted on the surface of PVA/CA composite membrane. With the increasing concentration of TiO_2_ nanoparticles, the nano-sized TiO_2_ particles tended to form aggregates and dispersed onto the PVA/CA membrane surface. Due to the hydroxyl functional groups (O-H) on the surface of PVA/CA composite membrane, the Ti^4+^ from TiO_2_ nanoparticles were bond with the oxygen forming O-H bonds of membrane structure. There are hydroxyl functional groups (O-H) on the structure of cross-linked PVA composite membrane. The TiO_2_ nanoparticles were difficult to be washed and removed from membrane surface, which could be ascribed to the self-assembly of TiO_2_ and the strong interaction between TiO_2_ and PVA polymer.

### Membrane flux experiments

**Figure 3:**
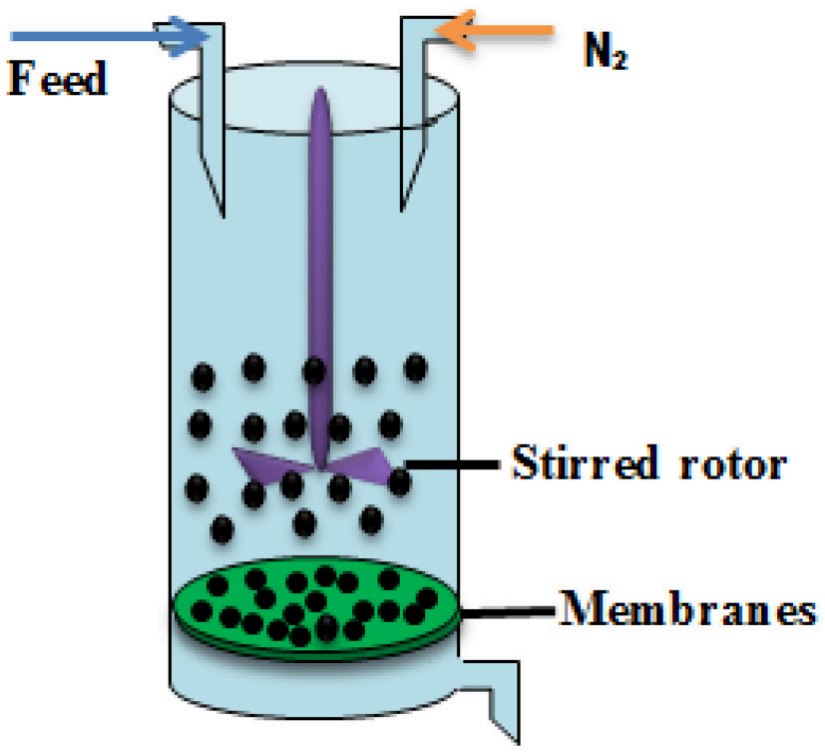
**The experimental filtration rig**.

**Figure 4:**
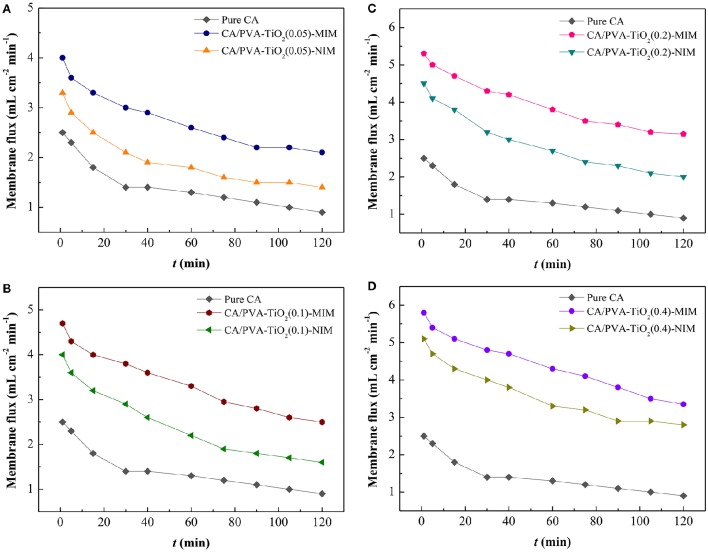
**Membrane flux of Pure CA and CA/PVA-TiO_**2**_(0.05%)-MIM/NIM (A)** ; Pure CA and CA/PVA-TiO_2_(0.1%)-MIM/NIM **(B)**; Pure CA and CA/PVA-TiO_2_(0.2%)-MIM/NIM **(C)**; Pure CA and CA/PVA-TiO_2_(0.4%)-MIM/NIM **(D)**. (The experiment was repeated three times, the data averaged. The standard deviations (sd) of all the data point are lower than 0.68).

To determine the hydrophilicity performance of membrane, the filtration experiments carried out in dead-end cells, and the experimental filtration rig was shown in Figure 3. The membrane flux was performed at the trans-membrane pressure of 0.15 Mpa (An et al., 2013; Meng et al., 2014; Shao et al., 2014) and the results were presented in Figure 4.

As shown in Figure 4A, the pure CA membrane has the lowest membrane flux, which could be ascribed to the denser membrane structure and less pores of pure CA membrane. In the Figures 4A–D, compared with the flux of pure CA membrane, CA/PVA-TiO_2_-MIM and CA/PVA-TiO_2_-NIM presented higher flux values, due to the hydrophilic TiO_2_ on membrane surface. Furthermore, the flux values were high at beginning and gradually declined with the increase of operation time, which could be ascribed to the pore structure and binding sites in membranes as for the results discussed in the SEM analysis. We suspected that the decrease of flux values was attributed to the jammed inner pores and SA adsorption. In addition, the CA/PVA-TiO_2_-MIM exhibited higher membrane flux than that of CA/PVA-TiO_2_-NIM at similar pressure. This result suggested that the CA/PVA-TiO_2_-MIM prepared by phase inversion imprinting technique and grafted by TiO_2_ was beneficial to the mass transport for SA-contained aqueous solution, which facilitated the recognition capability between membrane and SA molecules.

The modification of different amount of TiO_2_ nanoparticles on the CA/PVA composite membranes had brought changes to the membrane flux. As expected, the flux profiles of CA/PVA-TiO_2_(0.05%)-MIM/NIM, CA/PVA-TiO_2_(0.1%)-MIM/NIM, CA/PVA-TiO_2_(0.2%)-MIM /NIM and CA/PVA-TiO_2_(0.4%)-MIM/NIM were remarkably increased with the increasing concentration of grafted TiO_2_ (see Figures 4A–D). This was mainly due to the hydrophilic and antifouling of TiO_2_, which increased the interaction between water molecules and modified membranes. Consequently, the grafted TiO_2_ could easily enhance the permeate rate between the membrane and permeants, and thereby leading to high flux of the TiO_2_ modified CA/PVA composite membranes.

### Batch adsorption kinetics

**Figure 5:**
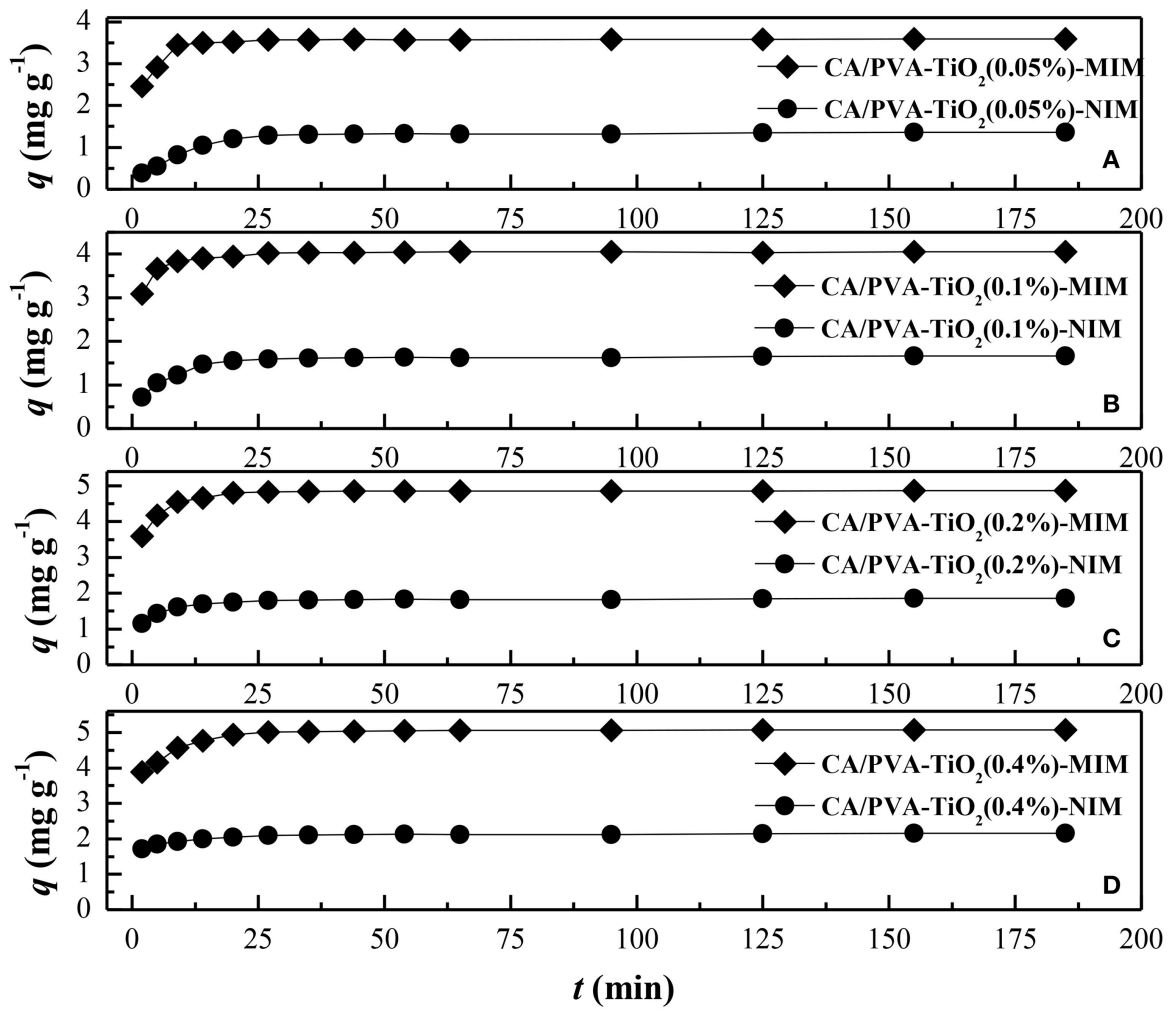
**Comparison of the adsorption kinetics of different membranes: (A)** CA/PVA-TiO_2_(0.05%)-MIM and NIM, **(B)** CA/PVA-TiO_2_(0.1%)-MIM and NIM, **(C)** CA/PVA-TiO_2_(0.2%)-MIM and NIM and **(D)** CA/PVA-TiO_2_(0.4%)-MIM and NIM. (The experiment was repeated three times, the data averaged. The standard deviations (sd) of all the data point are lower than 0.53).

The adsorption rate was a crucial parameter used to reflect the adsorption process. Figure 5 showed the adsorption kinetic curves of SA on MIM (CA/PVA-TiO_2_(0.05%, 0.1%, 0.2%, 0.4%)-MIM) and NIM (CA/PVA-TiO_2_(0.05%, 0.1%, 0.2%, 0.4%)-NIM) from methanol aqueous solution containing 100 mg L^−1^ SA at different sampling time. It could be seen that the adsorption equilibrium time of MIM and NIM were 15 and 30 min, respectively. It was reasonable to assume that a large amount of specific imprinting sites existed in MIM, thus it was easier for the template SA to bind with the imprinting sites and the MIM reached the adsorption equilibrium faster than NIM. With the template SA binding with the imprinting sites, the subsequent templates hardly be recognized by the sites under the effect of diffusion resistance, so that the adsorption rate of SA increase slowly and the reached equilibrium. In this study, it was found that the adsorption kinetic of CA/PVA-TiO_2_(0.2%)-MIM for SA was much higher than that of other corresponding imprinted membranes. It suggested that the templates SA were much easier to access the CA/PVA-TiO_2_(0.2%)-MIM than other membranes.

To further investigate the underlying mechanism of adsorption processes, the kinetics of SA adsorption on CA/PVA-TiO_2_(0.2%)-MIM and CA/PVA-TiO_2_(0.2%)-NIM were analyzed by two typical kinetic models: the pseudo-first-order (Equation 9) and pseudo-second-order rate equation (Equation 7) (Wu et al., 2014b). The pseudo-first-order model can be described in following equation:

(9) $$\begin{array}{l} q_{\text{t}}=q_{\text{e}}-q_{\text{e}}e^{-k_{1}t} \end{array}$$

where *q*_e_ and *q*_t_ (mg g^−1^) represent the adsorption capacity at equilibrium and any time *t* (min), respectively. *k*_1_ (min^−1^) present the pseudo-first-order rate constant of adsorption.

The pseudo-second-order model can be expressed as follows:

(10) $$\begin{array}{l} q_{t}=\frac{k_{2}q_{e}^{2}t}{1+k_{2}q_{e}t} \end{array}$$

*k*_2_ (g mg^−1^ min^−1^) present the pseudo-second-order rate constant of adsorption.

**Figure 6:**
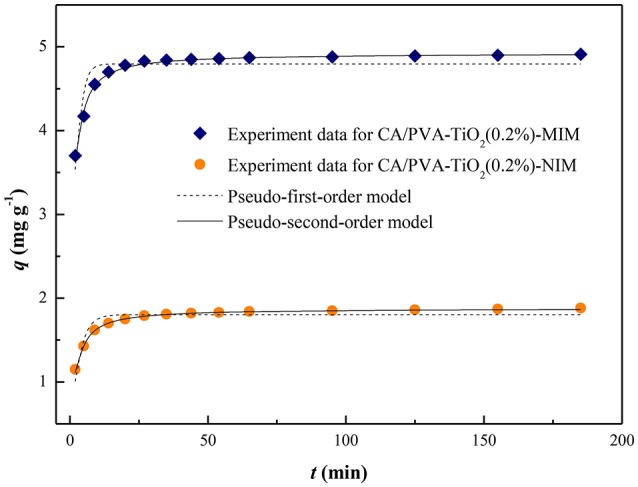
**The non-linear regression of kinetic models for CA/PVA-TiO_**2**_(0.2%)-MIM and CA/PVA-TiO_**2**_(0.2%)-NIM**.

In this study, the adsorption kinetic constants and non-linear regression values were listed in Table 1, and the non-linear regression plots of the two models for SA binding were presented in Figure 6. The correlation coefficient (*R*^2^) was used to judge the applicability of the kinetic models. As shown in Table 1, the correlation coefficient values (*R*^2^ values above 0.95) of the adsorption processed on CA/PVA-TiO_2_(0.2%)-MIM or CA/PVA-TiO_2_(0.2%)-NIM by pseudo-second-order kinetic model were higher than that by pseudo-first-order model. The pseudo-second-order model exhibited the favorable agreement between the theoretical *q*_e_ values (*q*_e, cal_) and the experimental *q*_e_ values (*q*_e, exp_), while the opposite result was found for the pseudo-first-order model. Therefore, the results suggested that the pseudo-second-order kinetic model was well-described the adsorption behavior of SA.

**Table 1:** **Kinetics constants for the pseudo-first-order and pseudo-second-order equations**.

**Adsorbents**	**Pseudo-first-order model**				**Pseudo-second-order model**		
	***q*_e, exp_ (mg g^−1^)**	***q*_e, cal_ (mg g^−1^)**	***k*_1_ (min^−1^)**	***R*^2^**	***q*_e, cal_ (mg g^−1^)**	***k*_2_(g mg^−1^ min^−1^)**	***R*^2^**
CA/PVA-TiO_2_(0.2%)-MIM	4.9113	4.7961	0.6684	0.7561	4.9235	0.2852	0.9816
CA/PVA-TiO_2_(0.2%)-NIM	1.8804	1.8028	0.4089	0.8248	1.8774	0.3878	0.9926

### Adsorption isotherm

**Figure 7:**
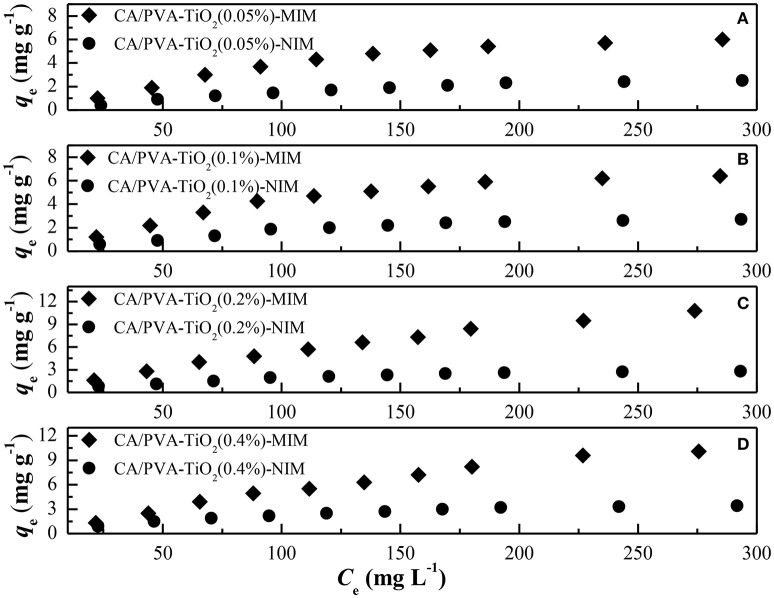
**Comparison of the adsorption isotherms of different membranes: (A)** CA/PVA-TiO_2_(0.05%)-MIM and NIM, **(B)** CA/PVA-TiO_2_(0.1%)-MIM and NIM, **(C)** CA/PVA-TiO_2_(0.2%)-MIM and NIM and **(D)** CA/PVA-TiO_2_(0.4%)-MIM and NIM. (The experiment was repeated three times, the data averaged. The standard deviations (sd) of all the data point are lower than 0.65).

To evaluate the adsorption performance of the MIM and NIM for SA, the adsorption isotherm experiments were carried out at room temperature. As shown in Figure 7, the equilibrium adsorption capacities increased gradually with the increase of the SA initial concentration, and ultimately reached an equilibrium value due to the saturation of static adsorption behavior. It can also be observed that the adsorption capacity of MIM was high than that of NIM, which might be ascribed to a great deal of chemical imprinting sites in MIM, so that SA molecules were more easily interacting with MIM. The NIM showed non-selective physical adsorption toward SA molecules. Furthermore, the adsorption capacity of CA/PVA-TiO_2_(0.2%)-MIM toward SA was much higher than that of CA/PVA-TiO_2_(0.05%)-MIM, CA/PVA-TiO_2_(0.1%)-MIM, CA/PVA-TiO_2_(0.2%)-MIM, and CA/PVA-TiO_2_(0.4%)-MIM. The results suggested that the CA/PVA-TiO_2_(0.2%)-MIM were the optimal imprinted membrane in the present work.

In this study, the equilibrium data for SA onto CA/PVA-TiO_2_(0.2%)-MIM and CA/PVA-TiO_2_(0.2%)-NIM were fitted by two classical isotherm models, i.e., The Langmuir model (Equation 11; Xue et al., 2009) and Freundlich model (Equation 12; Yi et al., 2011).

(11) $$\begin{array}{rc} q_{\text{e}} & =\frac{q_{\text{m}}K_{\text{L}}C_{\text{e}}}{1+K_{\text{L}}C_{\text{e}}} \end{array}$$

(12) $$\begin{array}{rc} q_{\text{e}} & =K_{\text{F}}C_{\text{e}}^{1/\text{n}} \end{array}$$

where *C*_e_ (mg L^−1^) represents the equilibrium concentration of SA. *q*_e_ (mg g^−1^) and *q*_m_ (mg g^−1^) represent the equilibrium amount and the maximum adsorption capacity of SA, respectively. *K*_L_ is the Langmuir constant related to the affinity of the adsorption sites. *K*_F_ and 1/*n* are Freundlich constants related to the capacity and intensity of the adsorption, respectively.

**Figure 8:**
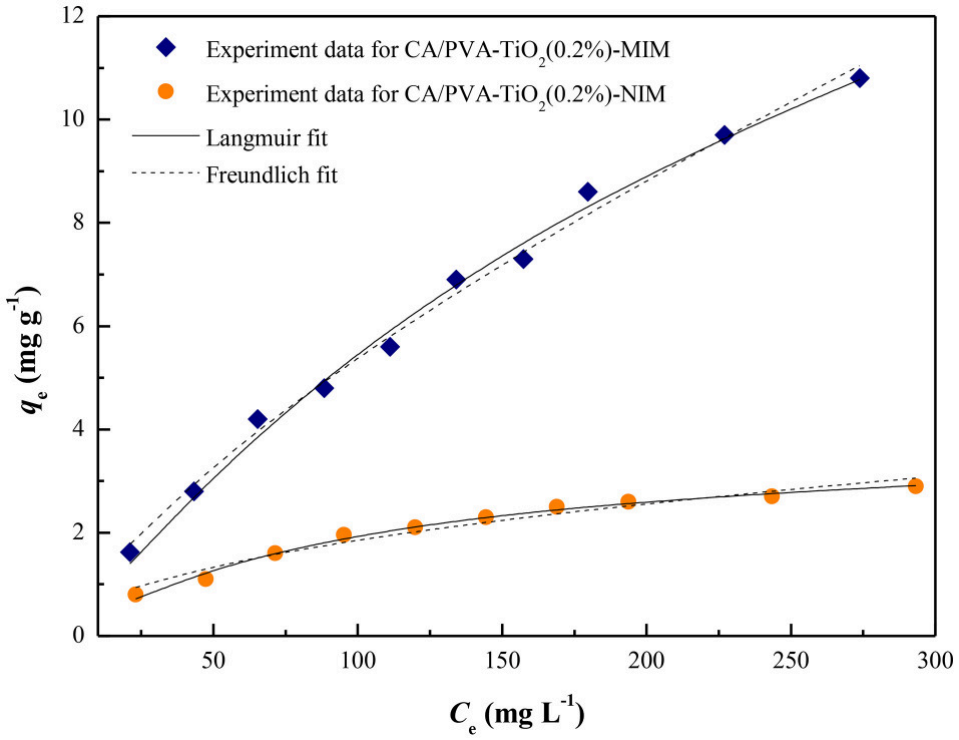
**Adsorption isotherms of SA on the CA/PVA-TiO_**2**_(0.2%)-MIM and CA/PVA-TiO_**2**_(0.2%)-NIM with the fitting to the Langmuir model and the Freundlich model**.

A comparison of the isotherm models for SA adsorption onto CA/PVA-TiO_2_(0.2%)-MIM and CA/PVA-TiO_2_(0.2%)-NIM with non-linear regression were shown in Figure 8 and the adsorption isotherm constants were given in Table 2. The correlation coefficient (*R*^2^) was used to judge the applicability of the isotherm models. As shown in Table 2, the experimental data were well-fitted by the two models (Langmuir and Freundlich models) with *R*^2^ values: 0.9932, 0.9921; 0.9899, 0.9587 for CA/PVA-TiO_2_(0.2%)-MIM and CA/PVA-TiO_2_(0.2%)-NIM, respectively. Apparently, the correlation coefficient was higher with the Langmuir model than with the Freundlich model, which indicated that the Langmuir model provided better fitting for CA/PVA-TiO_2_(0.2%)-MIM and CA/PVA-TiO_2_(0.2%)-NIM separately. The calculated maximum adsorption capacities of CA/PVA-TiO_2_(0.2%)-MIM and CA/PVA-TiO_2_(0.2%)-NIM were 24.4338 mg g^−1^ and 3.9295 mg g^−1^, respectively.

**Table 2:** **The adsorption isotherm constants for CA/PVA-TiO_**2**_(0.2%)-MIM and CA/PVA-TiO_**2**_(0.2%)-NIM at 25°C**.

**Adsorption isotherm models**	**Parameters**	**CA/PVA-TiO_2_(0.2%)-MIM**	**CA/PVA-TiO_2_(0.2%)-NIM**
Langmuir model	*R*^2^	0.9932	0.9899
	*K*_L_ (L mg^−1^)	0.0029	0.0097
	*q*_m, cal_ (mg g^−1^)	24.4338	3.9295
Freundlich model	*R*^2^	0.9921	0.9587
	*K*_F_ (mg g^−1^)	0.2022	0.2183
	*n*	1.4030	2.1521

In addition, the binding affinity and the theoretical binding site number for template of CA/PVA-TiO_2_(0.2%)-MIM was further evaluated by Scatchard analysis. The equation is as follows:

(13) $$\begin{array}{l} \frac{q}{C}=\frac{\left(q_{\text{max}}-q\right)}{K_{\text{d}}} \end{array}$$

where *q* (mg g^−1^) is the equilibrium adsorption capacity of SA on CA/PVA-TiO_2_(0.2%)-MIM, *q*_max_ (mg g^−1^) is the maximal adsorption capacity of CA/PVA-TiO_2_(0.2%)-MIM for SA, *C* (mg mL^−1^) is the SA concentration in the test solution, and *K*_d_ (mg mL^−1^) is the dissociation equilibrium constant.

**Figure 9:**
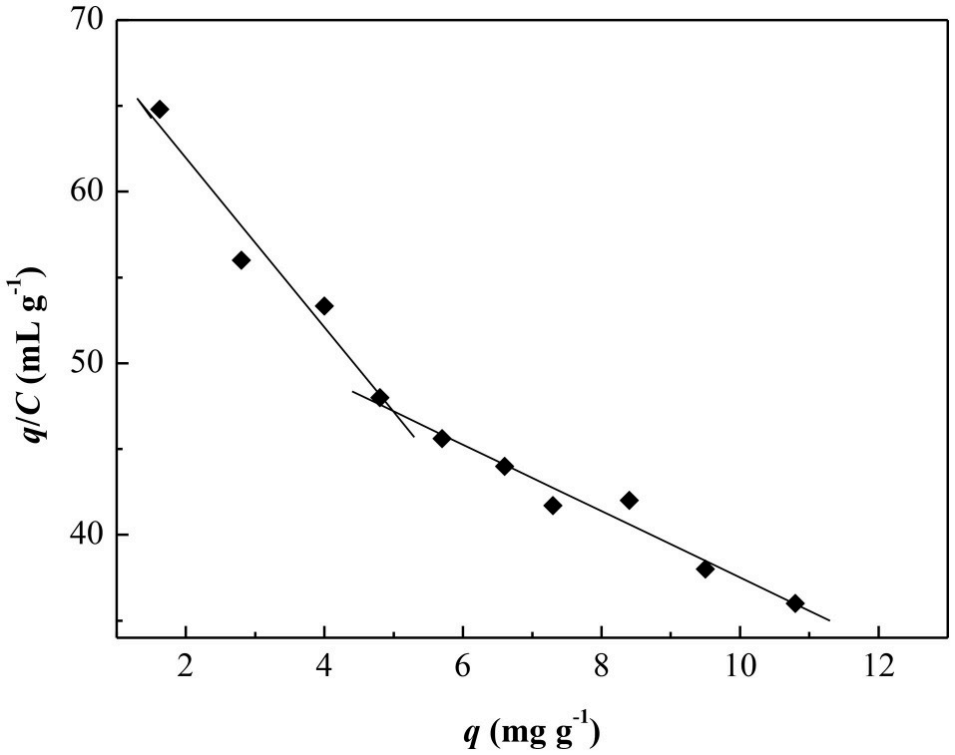
**Scatchard curve for CA/PVA-TiO_**2**_(0.2%)-MIM**.

Figure 9 showed the Scatchard plot of the adsorption of CA/PVA-TiO_2_(0.2%)-MIM for SA. The two distinct linear sections within the plot of *q*/*C* on the vertical axis and *C* on the horizontal axis were yielded by linear regression analysis. It was found that there existed two types of binding sites in respect to the affinity for SA of the CA/PVA-TiO_2_(0.2%)-MIM. As shown in Table 3, the two sets of binding parameters (*K*_d1_ = 0.20 mg mL^−1^, *q*_max1_ = 14.56 mg g^−1^ and *K*_d2_ = 0.52 mg mL^−1^, *q*_max1_ = 29.32 mg g^−1^) were obtained by the slopes and intercepts with the horizontal axis, which was corresponding to the high-affinity and low-affinity binding sites.

**Table 3:** **Results of the Scatchard analysis**.

**Binding site**	**Linear equation**	***K*_d_ (mg mL^−1^)**	***q*_max_ (mg g^−1^)**
Higher affinity sites	*q*/*C* = 71.82–4.93*q* (*R*^2^ = 0.9335)	0.20	14.56
Lower affinity sites	*q*/*C* = 56.88–1.94*q* (*R*^2^ = 0.9604)	0.52	29.32

### Selectivity binding

The selective adsorption ability of prepared membranes was evaluated toward competitive species SA, *p*-HB, and MS. The selective binding experiments for the similar compounds on different membranes were all carried out under same experimental conditions.

The results of distribution coefficient (*K*_*d*_) and selectivity coefficient of the sorbent (α) were summarized in Table 4. As shown in Table 4, the α-values of pure CA for SA relative to *p*-HB and MS were 0.96 and 0.94, respectively, indicating that pure CA was almost non-selectivity for SA. Table 4 also presented that the α-values of CA/PVA-TiO_2_(0.2%)-MIM for SA relative to *p*-HB and MS were higher (3.87 and 3.55, respectively), and the corresponding α of CA/PVA-TiO_2_(0.2%)-NIM were much lower (1.14 and 1.24, respectively). Obviously, the above fact fully displayed that MIM had stronger selective recognition ability than NIM, whereas the separation factor for *p*-HB was higher than that for MS.

**Table 4:** **Parameters of batch adsorption selectivity of prepared membranes**.

**Membrane**	***K*_d_ (L g^−1^)**		**α**
	**SA**	***p*-HB**	
Pure CA	0.01594	0.01659	0.9608
CA/PVA-TiO_2_(0.05%)-MIM	0.05130	0.01937	2.6484
CA/PVA-TiO_2_(0.1%)-MIM	0.06435	0.01768	3.6397
CA/PVA-TiO_2_(0.2%)-MIM	0.07005	0.01812	3.8659
CA/PVA-TiO_2_(0.2%)-NIM	0.04500	0.03955	1.1378
CA/PVA-TiO_2_(0.4%)-MIM	0.07205	0.01914	3.7644
	**SA**	**MS**	
Pure CA	0.01541	0.01635	0.9425
CA/PVA-TiO_2_(0.05%)-MIM	0.05062	0.02008	2.5209
CA/PVA-TiO_2_(0.1%)-MIM	0.05402	0.01690	3.1964
CA/PVA-TiO_2_(0.2%)-MIM	0.06647	0.01874	3.5470
CA/PVA-TiO_2_(0.2%)-NIM	0.04284	0.03453	1.2407
CA/PVA-TiO_2_(0.4%)-MIM	0.07073	0.02110	3.3521

Furthermore, the separation effect of MIM with various concentrations of TiO_2_ were evaluated and the α-values of CA/PVA-TiO_2_(0.05%), CA/PVA-TiO_2_(0.1%), CA/PVA-TiO_2_(0.2%), and CA/PVA-TiO_2_(0.4%) imprinted membranes for SA in relation to *p*-HB were 2.65, 3.64, 3.87, and 3.76, respectively, thus showed well-imprinting effect. This revealed that the grafted TiO_2_ may be beneficial to improve the effective adsorption sites in the CA blend imprinted membranes. Clearly, the α-value of CA/PVA-TiO_2_(0.2%)-MIM was highest among the prepared imprinted membranes, α-value of CA/PVA-TiO_2_(0.4%)-MIM was lower than that of CA/PVA-TiO_2_(0.2%)-MIM. It found that the MIM with large amount of TiO_2_ may not obtain a high selective recognition ability for SA. Excessive TiO_2_ on CA/PVA-TiO_2_(0.4%)-MIM may lead to limit the imprinting effect for SA with the increase of jammed pores. Consequently, the above results suggested that CA/PVA-TiO_2_(0.2%)-MIM could be selected as the optimal imprinted membrane in selective binding experiments. experiments.

### Regeneration and reproducibility of membranes

**Figure 10:**
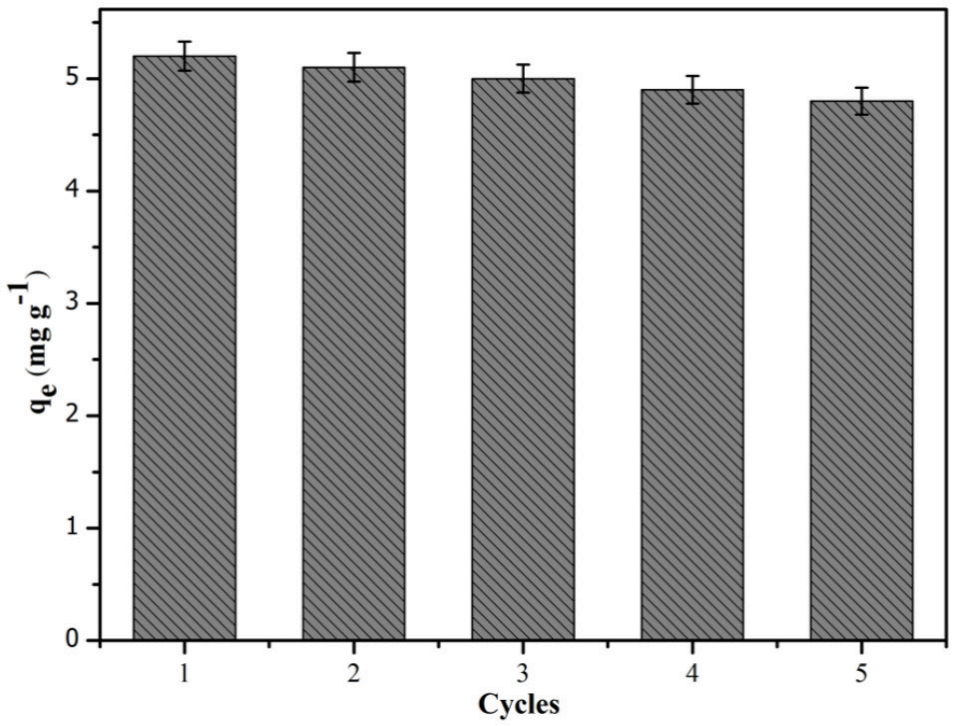
**Adsorption-desorption cycles for CA/PVA-TiO_**2**_(0.2%)-MIM**.

To evaluate the stability and regeneration of the CA/PVA-TiO_2_(0.2%)-MIM, the regeneration experiment was performed at the SA concentration of 100 mg L^−1^. After adsorption of SA onto CA/PVA-TiO_2_(0.2%)-MIM, the SA-adsorbed CA/PVA-TiO_2_(0.2%)-MIM was regenerated using the methanol/acetic acid (9:1, v/v) mixed solvent and deionized water. As shown in Figure 10, the adsorption-desorption experiment was repeated five times using the same CA/PVA-TiO_2_(0.2%)-MIM membrane. Obviously, the CA/PVA-TiO_2_(0.2%)-MIM could be effectively reused for five times with only about 9.17% decrease of initial binding capacity. This result suggested that the CA/PVA-TiO_2_(0.2%)-MIM could be reused at least five times without significant decrease in their adsorption capacities.

**Figure 11:**
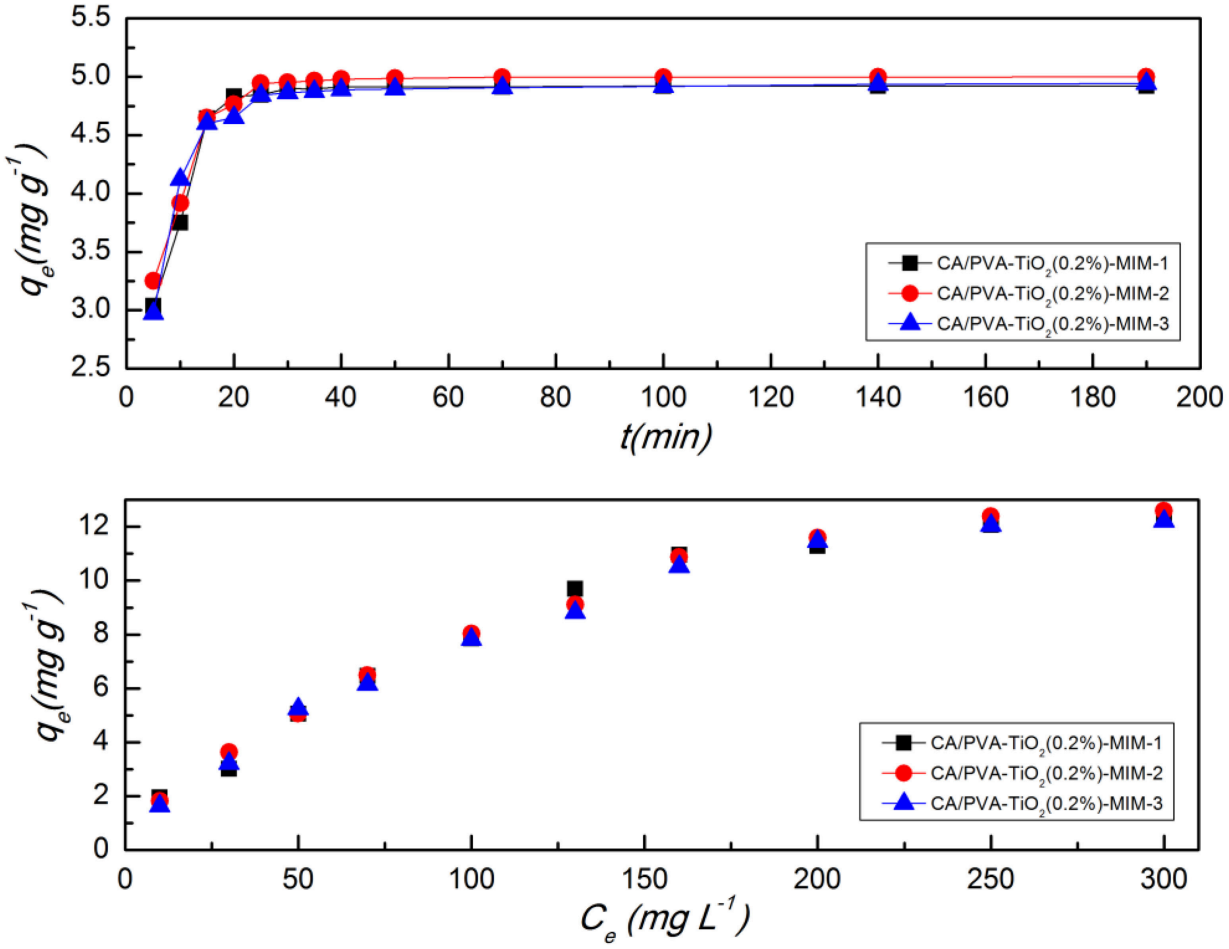
**Comparison of the adsorption isotherms and adsorption kinetics of three batches CA/PVA-TiO_**2**_(0.2%)-MIM**. CA/PVA-TiO_2_(0.2%)-MIM-1 is the first group, CA/PVA-TiO_2_(0.2%)-MIM-2 is the second group and CA/PVA-TiO_2_(0.2%)-MIM-3 is the third group. (The experiment was repeated three times, the data averaged. The standard deviations (sd) of all the data point are lower than 0.72).

In 3 weeks, the CA/PVA-TiO_2_(0.2%)-MIM was synthesized for three times, and the batch adsorption kinetics and the adsorption isotherm were studied in order to demonstrate its reproducibility of synthesis. The adsorption kinetics and adsorption isotherm of three batches were given in Figure 11, in which the first batch was named CA/PVA-TiO_2_(0.2%)-MIM-1, and so on. It was found that the adsorption properties of CA/PVA-TiO_2_(0.2%)-MIM was stable from Figure 11.

### Antifouling ability of membranes

**Figure 12:**
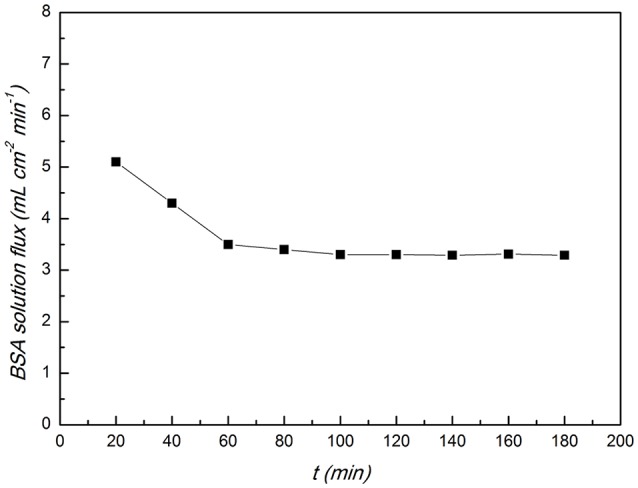
**BSA solution flux of the CA/PVA-TiO_**2**_(0.2%)-MIM**. (The experiment was repeated three times, the data averaged. The standard deviations (sd) of all the data point are lower than 0.69).

The CA/PVA-TiO_2_(0.2%)-MIM was tested by BSA solution filtration to evaluate its antifouling properties. Figure 12 showed how the BSA solution flux changes as a function of filtration time. The BSA solution flux decreased at the early stage of the filtration and kept constant after 60 min from the Figure 12. This was because that at the beginning of filtration the surface of the CA/PVA-TiO_2_(0.2%)-MIM absorbed some protein molecules and the BSA solution flux decreased. And then the rate of the attachment of protein molecules to the surface of the CA/PVA-TiO_2_(0.2%)-MIM was almost the same as the rate of the release of protein molecules from the surface, therefore, the BSA solution flux reached steady state.

As the key parameters for the evaluation of the fouling resistant properties of the membranes, the flux recovery ration (FRR), reversible fouling (*FR*_*r*_) and irreversible fouling (*FR*_*ri*_) was calculated according to the Equations 6–8. An membrane with higher antifouling performance will have a higher *FRR* and a lower *FR*_*r*_ and *FR*_*ri*_. The FRR of CA/PVA-TiO_2_(0.2%)-MIM reached 91%, and the *FR*_*r*_ and *FR*_*ri*_ are 24.3 and 9.0%. It indicated that the CA/PVA-TiO_2_(0.2%)-MIM had very good antifouling performances.

## Conclusions

In this work, a series of TiO_2_ grafted CA/PVA composite imprinted membranes for template SA were synthesized combining phase inversion technique and dip coating technique. The characteristics, membrane flux, kinetics, adsorption capacities, selectivity, and regeneration were studied in detail. Morphological analysis and membrane flux showed that the surface morphology and hydrophilicity were strongly improved with the concentration of grafted TiO_2_. The adsorption kinetic analysis suggested that the CA/PVA-TiO_2_(0.2%)-MIM was the optimal imprinted membrane and possessed high kinetic equilibrium adsorption capacity for the binding of SA. The adsorption capacity of CA/PVA-TiO_2_(0.2%)-MIM can reach above 4.8 mg g^−1^ after 25 min. The pseudo-second-order kinetic model was able to predict the adsorption behavior of SA on CA/PVA-TiO_2_(0.2%)-MIM and implied that the adsorption process was a chemical process. Moreover, adsorption isotherm analysis showed that the CA/PVA-TiO_2_(0.2%)-MIM had high binding capacity for SA. The limit binding capacity of CA/PVA-TiO_2_(0.2%)-MIM could meet 7.1 mg g^−1^ when the concentration of SA is 150 mg L^−1^. The selectivity binding results revealed that the CA/PVA-TiO_2_(0.2%)-MIM had excellent selective recognition ability for SA in respect to the competitive analogs. The α-values of CA/PVA-TiO_2_(0.2%)-MIM for SA relative to *p*-HB and MS were 3.87 and 3.55. The regeneration experiment exhibited that the CA/PVA-TiO_2_(0.2%)-MIM could be reused at least five adsorption-desorption cycles without decreasing the binding capacity significantly. The CA/PVA-TiO_2_-MIM had good performance of adsorption capacity, selectivity binding, regeneration and antifouling ability. The real wastewater had complex components, and further studies would be performed to extend this research to separate SA from complex matrix.

## Author contributions

XY and YY are in charge of the design for the imprinted membranes. ZH and MM are in charge of the synthesis of the imprinted membranes, and HL is in charge of the structural characterization of the imprinted membranes. XY and XM are in charge of the simulations and calculation in the experiment.

### Conflict of interest statement

The authors declare that the research was conducted in the absence of any commercial or financial relationships that could be construed as a potential conflict of interest.

## References

[B1] Abedini R. Mousavi S. M. Aminzadeh R. (2011). A novel cellulose acetate (CA) membrane using TiO2 nanoparticles: preparation, characterization and permeation study. Desalination 277, 40–45. 10.1016/j.desal.2011.03.089

[B2] Ahmad A. L. Abdulkarim A. A. Ooi B. S. Ismail S. (2013). Recent development in additives modifications of polyethersulfone membrane for flux enhancement. Chem. Eng. J. 223, 246–267. 10.1016/j.cej.2013.02.130

[B3] An Q. F. Ji Y. L. Hung W. S. Lee K. R. Gao C. J. (2013). AMOC positron annihilation study of zwitterionic nanofiltration membranes: correlation between fine structure and ultrahigh permeability. Macromolecules 46, 2228–2234. 10.1021/ma400193s

[B4] Cheku M. F. Yusuke H. Takaomi K. (2008). Scaffold membranes for selective adsoption of a-tocopherol by phase inversion covalenty imprinting technique. J. Membr. Sci. 322, 503–501. 10.1016/j.memsci.2008.05.046

[B5] Chen W. Ma Y. Pan J. M. Meng Z. H. Pan G. Q. Sellergren B. (2015). Molecularly imprinted polymers with stimuli-responsive affinity: progress and perspectives. Polymers 7, 1689–1715. 10.3390/polym7091478

[B6] Del Blanco S. G. Donato L. Drioli E. (2012). Development of molecularly imprinted membranes for selective recognition of primary amines in organic medium. Sep. Purif. Technol. 87, 40–46. 10.1016/j.seppur.2011.11.018

[B7] Dima S. O. Meouche W. Dobre T. Nicolescu T. V. Sarbu A. (2013). Diosgenin-selective molecularly imprinted pearls prepared by wet phase inversion. React. Funct. Polym. 73, 1188–1197. 10.1016/j.reactfunctpolym.2013.05.014

[B8] Donato L. Tasselli F. De Luca G. Del Blanco G. S. Drioli E. (2013). Novel hybrid molecularly imprinted membranes for targeted 4,4′-methylendianiline. Sep. Purif. Technol. 116, 184–191. 10.1016/j.seppur.2013.05.027

[B9] Ghaemi N. Madaeni S. S. Alizadeh A. Daraei P. Vatanpour V. Falsafi M. (2012). Fabrication of cellulose acetate/sodium dodecyl sulfate nanofiltration membrane: characterization and performance in rejection of pesticides. Desalination 290, 99–106. 10.1016/j.desal.2012.01.013

[B10] Han R. L. Zeng J. H. Wang Y. Q. Chang Q. B. Zhang X. Z. Zhou J. (2014). Preparation and application of positively charged quaternized chitosan/PEI composite nanofiltration membranes. Desalin. Water Treat. 52, 5790–5795. 10.1080/19443994.2013.817373

[B11] He Z. H. Meng M. J. Yan L. Zhu W. H. Sun F. Q. Yan Y. S. (2015). Fabrication of new cellulose acetate blend imprinted membrane assisted with ionic liquid ([BMIM]Cl) for selective adsorption of salicylic acid from industrial waste water. Sep. Purif. Technol. 145, 63–74. 10.1016/j.seppur.2015.03.005

[B12] Huang J. Hu Y. Hu Y. Li G. (2013). Disposable terbium (III) salicylate complex imprinted membrane using solid phase surface fluorescence method for fast separation and detection of salicylic acid in pharmaceuticals and human urine. Talanta 107, 49–54. 10.1016/j.talanta.2012.12.054 23598191

[B13] Jafari M. T. Badihi Z. Jazan E. (2012). A new approach to determine salicylic acid in human urine and blood plasma based on negative electrospray ion mobility spectrometry after selective separation using a molecular imprinted polymer. Talanta 99, 520–526. 10.1016/j.talanta.2012.06.023 22967588

[B14] Luo X. Guo B. Luo J. Deng F. Zhang S. Luo S. (2015). Recovery of lithium from wastewater using development of Li ion-imprinted polymers. ACS Sustain. Chem. Eng. 3, 460–467. 10.1021/sc500659h

[B15] Meng M. Feng Y. Liu Y. Wang Y. Yan Y. (2014). Preparation of composite-imprinted alumina membrane for effective separation of p-hydroxybenzonic acid from its isomer using box-behnken design-based statistical modeling. J. Appl. Polym. Sci. 131, 590–600. 10.1002/app.40621

[B16] Meng M. J. Feng Y. H. Zhang M. Ji Y. J. Dai J. D. Liu Y. (2013). Optimization of surface imprinted layer attached poly(vinylidene fluoride) membrane for selective separation of salicylic acid from acetylsalicylic acid using central composite design. Chem. Eng. J. 231, 132–145. 10.1016/j.cej.2013.07.015

[B17] Pan G. Zhang Y. Ma Y. Li C. Zhang H. (2011). Efficient one-pot synthesis of water-compatible molecularly imprinted polymer microspheres by facile RAFT precipitation polymerization. Angew. Chem. 50, 11731–11734. 10.1002/anie.201104751 21990099

[B18] Peng Y. L. Dong Y. J. Fan H. W. Chen P. Li Z. H. Jiang Q. (2013). Preparation of polysulfone membranes via vapor-induced phase separation and simulation of direct-contact membrane distillation by measuring hydrophobic layer thickness. Desalination 316, 53–66. 10.1016/j.desal.2013.01.021

[B19] Puoci F. Scoma A. Cirillo G. Bertin L. Fava F. (2012). Selective extraction and purification of gallic acid from actual site olive mill wastewaters by means of molecularly imprinted microparticles. Chem. Eng. J. 198–199, 529–535. 10.1016/j.cej.2012.05.095

[B20] Rana D. Scheier B. Narbaitzb R. M. Matsuuraa T. Tabe S. Jasim S. Y. (2012). Comparison of cellulose acetate (CA) membrane and novel CA membranes containing surface modifying macromolecules to remove pharmaceutical and personal care product micropollutants from drinking water. J. Membrane Sci. 409–410, 346–354. 10.1016/j.memsci.2012.04.005

[B21] Razali M. Kim J. F. Attfield M. Budd P. M. Drioli E. Lee Y. M. (2015). Sustainable wastewater treatment and recycling in membrane manufacturing. Green Chem. 17, 5196–5205. 10.1039/C5GC01937K

[B22] Sellergren B. (2000). Molekular geprägte polymere mit einem gedächtnis für kleine moleküle, proteine oder kristalle. Angew. Chem. 112, 1071–1078. 10.1002/(sici)1521-3757(20000317)112:6<1071::aid-ange1071>3.0.co;2-x

[B23] Shao L. L. An Q. F. Ji Y. L. Zhao Q. Wang X. S. Zhu B. K. (2014). Preparation and characterization of sulfated carboxymethyl cellulose nanofiltration membranes with improved water permeability. Desalination 338, 74–83. 10.1016/j.desal.2014.01.025

[B24] Székely G. Valtcheva I. B. Kim J. F. Livingston A. G. (2015). Molecularly imprinted organic solvent nanofiltration membranes - Revealing molecular recognition and solute rejection behaviour. React. Funct. Polym. 86, 215–224. 10.1016/j.reactfunctpolym.2014.03.008

[B25] Wang C. Hu X. Guan P. Wu D. Qian L. Li J.. (2015). Separation and purification of thymopentin with molecular imprinting membrane by solid phase extraction disks. J. Pharmaceut. Biomed. Anal. 102, 137–143. 10.1016/j.jpba.2014.07.016 25265188

[B26] Wang H. Y. Kobayashi T. Fujii N. (1996). Molecular imprint membranes prepared by the phase inversion technique. Langmuir 12, 4850–4856. 10.1021/la960243y

[B27] Wang J. Qiu H. Shen H. Pan J. Dai X. Sellergren B. (2016). Molecularly imprinted fluorescent hollow nanoparticles as sensors for rapid and efficient detection λ-cyhalothrin in environmental water. Biosens. Bioelectron. 85, 387–394. 10.1016/j.bios.2016.05.041 27208472

[B28] Wu Y. L. Meng M. J. Liu X. L. Li C. X. Zhang M. Ji Y. J. (2014b). Efficient one-pot synthesis of artemisinin-imprinted membrane by direct surface-initiated AGET-ATRP. Sep. Purif. Technol. 131, 117–125. 10.1016/j.seppur.2014.05.001

[B29] Wu Y. L. Yan Y. S. Pan J. M. Dai X. H. Shi W. D. Meng M. J. (2014a). Fabrication and evaluation of molecularly imprinted regenerated cellulose composite membranes via atom transfer radical polymerization. Chinese Chem. Lett. 25, 273–278. 10.1016/j.cclet.2013.11.019

[B30] Xue Y. J. Hou H. B. Zhu S. J. (2009). Adsorption removal of reactive dyes from aqueous solution by modified basic oxygen furnace slag: isotherm and kinetic study. Chem. Eng. J. 147, 272–279. 10.1016/j.cej.2008.07.017

[B31] Yi X. S. Shi W. X. Yu S. L. Wang Y. Sun N. Jin L. M.. (2011). Isotherm and kinetic behavior of adsorption of anion polyacrylamide (APAM) from aqueous solution using two kinds of PVDF UF membranes. J. Hazard. Mater. 189, 95–501. 10.1016/j.jhazmat.2011.02.063 21398032

[B32] Zafar M. Ali M. Khan S. M. Jamil T. M. Butt T. Z. (2012). Effect of additives on the properties and performance of cellulose acetate derivative membranes in the separation of isopropanol/water mixtures. Desalination 285, 359–365. 10.1016/j.desal.2011.10.027

[B33] Zhang Y. Q. Shan X. Gao X. Q. (2011). Development of a molecularly imprinted membrane for selective separation of flavonoids. Sep. Purif. Technol. 76, 337–344. 10.1016/j.seppur.2010.10.024

[B34] Zhao X. Y. Ma J. Wang Z. H. Wen G. Jiang J. Shi F. M. (2012). Hyperbranched-polymer functionalized multi-walled carbon nanotubes for poly (vinylidenefluoride) membranes: from dispersion to blended fouling-control membrane. Desalination 303, 29–38. 10.1016/j.desal.2012.07.009

